# On Controversial Communications

**Published:** 1808-04

**Authors:** 


### *To the Editors of the Medical and Physical Journal.*

GENTLEMEN,

IN addressing you on a subject of equal delicacy and importance, it shall be my endeavour, as much as possible, to steer clear from the charges of personality, in order, by practice and example, to give additional force to the observations now submitted to your candor; and prove that the honor of medicine is of too much importance to be tampered with as a subject of fruitless and petulant animadversion. I shall likewise endeavour, by these remarks, to convince your readers that I am no advocate for individual unanimity of opinion on every subject relative to medicine, but, on the contrary, conceive that a diversity of sentiment is absolutely necessary to the improvement of art, the detection of error, and the advancement of science; and whilst I deprecate that popular opinion should in any degree become subservient to private maxims, or individual individual experience, I boldly contend that every man ought to be allowed the rightful prerogative of his *own* judgment, without exposing himself to the censure of those with whom he may not happen to coalesce. But, whatever advantage, or utility, it may be productive of when regulated solely by the dictates of propriety and decorum; yet, when it becomes unbridled by any restriction, instead of tending to enlarge the mind and instruct the judgment, it is only calculated to promote the exercise of individual tyranny and private injustice. It is, however, the business of a writer, who professes to aim at reformation, specifically to point out the errors and improprieties he deprecates, and to lay down plain and simple regulations for its future improvement, that whilst he is strenuously endeavouring to eradicate the former, he may be equally successful to enforce and establish the latter: with this view, therefore, Gentlemen, I must beg leave to solicit the exercise of your candor whilst I attempt to suggest a few fugitive observations, on some characteristic points observable in the late controversial communications of many of your correspondents, and which, in my opinion, appear equally unwarrantable and impolite.

Whatever may be the views or sentiments of a medical writer, when treating on controversial topics; or, however he may be influenced in support of his respective tenets, it affords no plea why he should couch his observations in that *asperity of language* so frequently manifested, and too generally adopted. He ought, rather as a duty he owes his character as a gentleman, and the profession as an individual member of it, to observe on all occasions that moderation, which, whilst it is equally calculated to carry force and impress conviction, is considerably more adapted to insure the esteem, and conciliate the respect of even those with whom he differs in sentiment. But error has here been successful to insinuate, that in order more effectually to wreath the palm of triumph over their opponents, it is necessary to adopt that asperity of style which now becomes the topic of my animadversion; but, let it be remembered, that this species of conduct, instead of tending to enhance their characters, or aggrandize their credit in the eyes of a medical and discerning public, is looked merely as a vulgar bravado! alike destitute of good manners and common sense.

Controversy has likewise become a fashionable vehicle for the advancement of *illiberal reflections*. Instead of being directed solely to the detection of erroneous notions, the the removal of speculative theories, and the establishment of genuine experience, it teems with the disgusting effusions of spleen, and groans under the enormous weight of personal invective; for whenever the opinions of opposing writers can neither be amicably reconciled or forcibly controverted, modern but vulgar practice disingenuously insinuates, that, in order to evade the opprobrium of cowardice, they must be ostentatiously ridiculed. Since, therefore, these observations are not the flighty excursions of fancy or chimerical product of speculation, but the genuine result of daily observation, I appeal to any impartial mind open to conviction, and unbiassed by prejudice, to decide whether they are characteristics calculated to dignify the conduct, and promote the reputation of men of science. To such a query, I hesitate not to anticipate a reply; duly impressed with a sense of the instinctive importance and professional responsibility of medicine, he will not only candidly allow, but feelingly deprecate, that a community who professedly disavow every sordid and mercenary view, to devote themselves wholly to the benefit of mankind, and who, on all other occasions, can observe with unlimited accuracy the most minute punctilios of etiquette, should, by illiberality of sentiment, couched in scurrility of language, at once degrade the honor of their profession, and forfeit every claim to the character of gentlemen, scholars, or christians.

But, in order to "lay the axe to the ROOT of the tree," and level our observations more directly to the ORIGIN of these illiberal practices, I believe every candid reader will allow the necessity of adverting to the *indulgence of unwarrantable tempers.* Perfectly indifferent as to the popular reception of the sentiment, I venture to affirm, that genuine and well-regulated controversy disowns, as unnecessary and poisonous ingredients, the insinuations of spleen, the effusions of envy, the emotions of petulance, or the aspersions of resentment. If, therefore, gentlemen, instead of the indulgence of these uncharitable dispositions, which so frequently convert the subjects of general attention and public investigation into topics of private animadversion and individual contest, your correspondents, whilst they zealously support their respective opinions, would evince more of the "milk of human kindness," so admirably calculated to adorn the conduct of human nature, how much better adapted would their communications be, to stand the brunt of every calumniating voice, and defy the decisive test of public scrutiny? As it is not my my intention to make any personal application of these general remarks, I shall only take the liberty to observe, that a gentleman, who stands foremost in the list of controversial writers, who ranks at the head of a public institution, and who has ever entered (perhaps too warmly) into the defence of a novel discovery; has, with a disposition, which I would almost call prejudice, injuriously attacked the character of many a private individual; and it is to be wished that the conduct of a gentleman, who of late, unfortunately became the subject of his illiberal insinuations, was more generally and successfully imitated. Aware, that by such a practice, not only private character but public reputation were at stake, he treated it not with a vulgar return of "railing for railing;" not with a disgraceful interchange of scurrilous aspersions; but with silent contempt.

I am, &c.

ALIQUIS.

*March* 10, 1808.

